# Impact of spinal or epidural anaesthesia on perioperative outcomes in adult noncardiac surgery: a narrative review of recent evidence

**DOI:** 10.1016/j.bja.2024.04.044

**Published:** 2024-05-28

**Authors:** David W. Hewson, Tiffany R. Tedore, Jonathan G. Hardman

**Affiliations:** 1 Department of Anaesthesia and Critical Care, Queen's Medical Centre, Nottingham University Hospitals NHS Trust, Nottingham, UK; 2 Academic Unit of Injury, Recovery and Inflammation Sciences, School of Medicine, University of Nottingham, Nottingham, UK; 3 Department of Anesthesiology, Weill Cornell Medicine, New York-Presbyterian Hospital/Weill Cornell Medical Center, New York, NY, USA

**Keywords:** epidural anaesthesia, general anaesthesia, patient-reported outcome measures, perioperative care, spinal anaesthesia

## Abstract

Spinal and epidural anaesthesia and analgesia are important anaesthetic techniques, familiar to all anaesthetists and applied to patients undergoing a range of surgical procedures. Although the immediate effects of a well-conducted neuraxial technique on nociceptive and sympathetic pathways are readily observable in clinical practice, the impact of such techniques on patient-centred perioperative outcomes remains an area of uncertainty and active research. The aim of this review is to present a narrative synthesis of contemporary clinical science on this topic from the most recent 5-year period and summarise the foundational scholarship upon which this research was based. We searched electronic databases for primary research, secondary research, opinion pieces, and guidelines reporting the relationship between neuraxial procedures and standardised perioperative outcomes over the period 2018–2023. Returned citation lists were examined seeking additional studies to contextualise our narrative synthesis of results. Articles were retrieved encompassing the following outcome domains: patient comfort, renal, sepsis and infection, postoperative cancer, cardiovascular, and pulmonary and mortality outcomes. Convincing evidence of the beneficial effect of epidural analgesia on patient comfort after major open thoracoabdominal surgery outcomes was identified. Recent evidence of benefit in the prevention of pulmonary complications and mortality was identified. Despite mechanistic plausibility and supportive observational evidence, there is less certain experimental evidence to support a role for neuraxial techniques impacting on other outcome domains. Evidence of positive impact of neuraxial techniques is best established for the domains of patient comfort, pulmonary complications, and mortality, particularly in the setting of major open thoracoabdominal surgery. Recent evidence does not strongly support a significant impact of neuraxial techniques on cancer, renal, infection, or cardiovascular outcomes after noncardiac surgery in most patient groups.

**Editor’s key points**

• Spinal and epidural blocks remain effective techniques in anaesthesia and acute pain management, even after the advent of minimally invasive surgery, fast-track programmes and fascial plane blocks.
• This narrative review summarises recent (2018-2023) evidence about patient-centred outcomes associated with spinal and epidural anaesthesia and analgesia.
• A beneficial effect of epidural analgesia on pain scores after major open thoracic and abdominal surgery outcomes was identified.
• The evidence of benefit in the prevention of cardiovascular, pulmonary, renal, infection, cancer, cognitive and morality outcomes was less consistent.
• Future clinical trials and subsequent meta-analyses on neuraxial techniques will benefit from more structured inclusion of standardised patient outcomes.

Subarachnoid and epidural anaesthesia were first described by August Bier in 1898 and Fidel Pagés in 1921, respectively.^1^ A century of scientific enquiry examining the anatomy, physiology, and pharmacology associated with neuraxial block, together with refinement of insertional techniques and procedural equipment, has given anaesthetists an excellent appreciation of their potential role, conduct, and limitations. Research delivered at a national scale has carefully defined the rare but potentially catastrophic complications of these procedures, allowing clinicians and patients to select specific techniques with informed discussion of material risk and benefit.^2^ The manifest anaesthetic effects, analgesic effects, or both of appropriately conducted epidural and spinal anaesthesia are readily observable in clinical practice and therefore these techniques have an important place in the armamentarium of many clinicians. Addressing what impact, if any, neuraxial block has on patient-centred outcomes beyond the immediate anaesthetic/analgesic effect accounts for a significant body of ongoing perioperative research, and this interest has coincided with new consensus-derived standardised definitions of perioperative outcome measures.^3^ Such outcome measures have been proposed as having primary importance to patients, clinicians, and health service providers.^4^ Careful assessment of the relationship between neuraxial techniques and standardised outcome measures will assist clinicians and patients in shared decision-making before surgery. Benefits often ascribed to neuraxial techniques (e.g. improved postoperative respiratory function), are increasingly debated as minimally invasive surgical approaches replace traditional, open surgery.

To summarise, appraise, and synthesise the most recent international evidence relating to the impact of spinal and epidural anaesthesia and analgesia on perioperative outcomes, we conducted a review of the literature published within the past 5 years, accompanied by narrative synthesis of results. Our aim is to describe the current state of evidence relating to the clinical effectiveness of neuraxial techniques applied to adult patients undergoing noncardiac surgery and to place this contemporary research in the context of preceding scholarship.

## Methods

In this review, we sought to identify contemporary articles describing spinal or epidural anaesthesia. After a literature search, we used narrative synthesis to summarise this contemporary research (defined as published within the past 5 years), alongside the historical studies upon which this science is based.

### Eligibility criteria

Inclusion criteria for studies were: (1) primary (observational or non-experimental studies, prospective trials) or secondary research (systematic reviews with or without meta-analyses), opinion pieces (editorial), or consensus-derived practice guidelines; (2) addressing perioperative outcomes from spinal or epidural analgesia or anaesthesia, either as single-shot block or catheter-based techniques, alone or in combination with general anaesthesia; (3) reported in non-pregnant adult (age ≥18 yr) human subjects undergoing noncardiac surgery; and (4) available in English language or with English language translation.

Exclusion criteria were primary study addressing the basic science relating to anatomy, procedural conduct, physiological consequences, or pharmacology of neuraxial block.

### Search strategy

Applying the principles described by Bramer and colleagues,^5^ we devised a search strategy and performed a search of MEDLINE and EMBASE for titles, abstracts, and keyword medical subject headings (MeSH) terms from electronic journal databases using the OVID platform between January 1, 2018 and September 1, 2023. Forty key general and relevant specialty journal databases were included in the search (Supplementary material). A grey literature search was conducted on the ProQuest thesis and dissertation platform. A supplementary search of Google Scholar was performed using the same date range. To minimise bias in the return of Google Scholar search results, that search was conducted using private web-browsing mode. The first 200 Google Scholar records were reviewed for inclusion. The following search terms were applied to titles and abstracts: spinal, intrathecal, subarachnoid, epidural, extradural, caudal, analgesia, anaesthesia. Terms were applied with spelling wildcards and Boolean operators (Supplementary material). After automated exclusion of duplicate entries, the titles and abstracts of returned studies were screened to determine whether they addressed perioperative outcome measures and should proceed to full-text review. Both initial screening and full-text review were performed by two authors (DWH and JGH), with the third author (TT) available to adjudicate if required. Articles addressing anatomical, procedural, physiological, or pharmacological considerations without consideration of perioperative outcome measures were excluded. The reference lists of studies that were selected for full-text review were screened for additional articles not identified via the above search strategy.

### Outcome measures of interest

Identified articles were assessed for their reporting of outcome measures established by the Standardised Endpoints in Perioperative Medicine (StEP) initiative^3^ according to the following domains: patient comfort and patient-centred outcomes^6^^,^^7^; infection and sepsis^8^; postoperative cancer outcomes^9^; renal endpoints^10^; cardiovascular outcomes^11^; pulmonary complications^12^; and mortality, morbidity, and organ failure.^13^ Table 1 provides a description of the identified StEP domains and core outcomes.

**Table 1:** Summary of published outcome measures advocated by the Standardised Endpoints in Perioperative Medicine (StEP) initiative. ∗Qualified recommendations or conditional endorsement.

StEP domain	Sub-domains	Recommended time point(s) for assessments	Recommended assessment tool(s)/definition or other comments
Patient comfort	Pain intensity at rest and during movement at 24 h after surgery	At 24 h after surgery and ideally at one other timepoint	11-point numeric rating scale
	Nausea and vomiting	0–6 h; 6–24 h and overall	Incidence of nausea and vomiting and use of rescue antiemetic medication
	Quality of recovery from anaesthesia and surgery		Quality of Recovery score or Quality of Recovery-15 scale
	Time to gastrointestinal recovery		Time to tolerate oral diet
	Time to mobilisation		
	Sleep disturbance		Patient-reported outcome measurement information system (PROMIS)-derived five-item Likert scale
Patient-centred outcomes	Health-related quality of life	12 months after surgery plus or minus 6-month assessment	EuroQol 5 dimension (EQ-5D-5L)
	Measurement of functional status		World Health Organization Disability Assessment Schedule version 2.0, 12-question version
	Life-impact measures	At 30 days after surgery plus or minus at 1 yr after surgery∗	Days alive and out of hospital
			Discharge destination∗
	Patient satisfaction	Within 24 h of surgery to a maximum of 48 h	Bauer patient satisfaction measure∗
Infection and sepsis	Fever	More than 24 h after surgery and two readings within a 12-h period	Core temperature >38.5°C
	Respiratory infectious complication	Within 30 days of surgery	Center for Disease Control definition
	Neurological infectious complication	Within 30 days of surgery	Center for Disease Control definition
	Urinary system infectious complication	Within 30 days of surgery	Center for Disease Control definition
	*Clostridium difficile* colitis/infection	Within 30 days of surgery	Center for Disease Control definition
	Endometritis	Within 30 days of surgery	Center for Disease Control definition
	Identification of pathogenic organism from tissue or fluid	Within 30 days of surgery	Based on Center for Disease Control definition
	Surgical site infection (SSI): superficial, deep, and/or organ/space	Within 30 days of surgery plus or minus within 90 days of surgery for deep and/or organ/space SSI in specific surgical subsets including breast, cardiac, spinal surgery	Center for Disease Control definitions
	Sepsis	Within 30 days of surgery	Third International Consensus Definitions for Sepsis and Septic Shock (SEPSIS-3)
	Septic shock	Within 30 days of surgery	Third International Consensus Definitions for Sepsis and Septic Shock (SEPSIS-3)
Postoperative cancer outcomes	Cancer health-related quality of life		‘A cancer related quality of life instrument’
	Days alive and out of hospital	At 90 days after surgery	
	Time to tumour progression		Time elapsed between treatment and tumour progression or death from tumour or cancer therapy
	Disease-free survival		Time elapsed between treatment and tumour progression or death from any cause
	Cancer-specific survival		Time elapsed between treatment and death from specific cancer
	Overall survival		Time elapsed between trial randomisation and death of any cause
Renal	Acute kidney injury		Kidney Disease: Improving Global Outcomes (KDIGO) consensus criteria, including or excluding oliguric criteria
	Acute kidney disease	At 30 days after surgery	≥30% decline in estimated glomerular filtration rate from baseline in patients meeting the creatine-based KDIGO acute-kidney-injury criteria within 7 days of surgery
	Composite of death or renal replacement therapy	Preferably 30 or 90 days after surgery	
	Major adverse kidney events (MAKE) composite	e.g. 30 plus or minus 90 days after surgery	Mortality or renal replacement therapy of any duration or ≥30% decline in estimated glomerular filtration rate from baseline
Cardiovascular	Myocardial infarction		StEP expert panel consensus-derived definition
	Myocardial injury		Fourth Universal Definition of Myocardial Infarction definition
	Cardiovascular death		StEP expert panel consensus-derived definition
	Nonfatal cardiac arrest		StEP expert panel consensus-derived definition
	Coronary revascularisation	Within 30 days of surgery	StEP expert panel consensus-derived definition
	Major adverse cardiac events		StEP expert panel consensus-derived definition
	Pulmonary embolism		StEP expert panel consensus-derived definition
	Deep vein thrombosis		StEP expert panel consensus-derived definition
	Atrial fibrillation		New onset of irregularly irregular heart rate in the absence of P waves lasting at least 30 s or for the duration of the ECG recording (if <30 s)
Pulmonary	Composite postoperative pulmonary complications		StEP expert panel consensus-derived definition
	Pneumonia		Center for Disease Control definition
	Respiratory failure		Berlin definition of respiratory distress syndrome
	Re-institution of mechanical ventilation	Within 30 days of surgery or for more than 24 h after surgery	The need for tracheal re-intubation and mechanical ventilation after extubation. The inclusion of non-invasive ventilation may be considered on a study-by-study basis.
Mortality, morbidity, and organ failure	Mortality	Within 30 days and 1 yr of surgery	
	Morbidity		Clavien–Dindo classification∗

## Results

**Fig 1:**
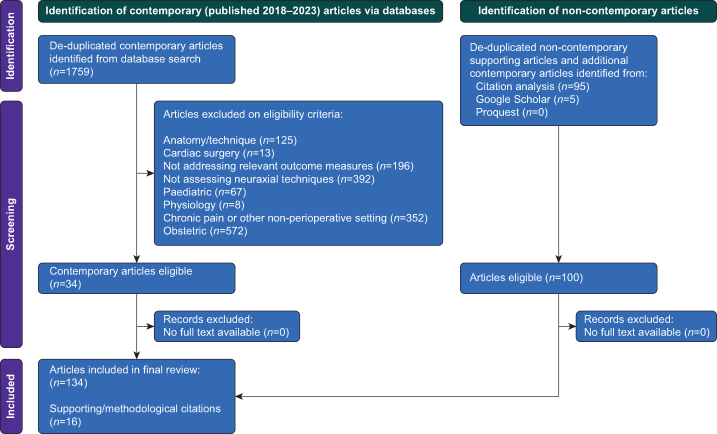
Flow diagram of search results.

A flow diagram of search results is shown in Figure 1. Our search strategy included caudal anaesthesia, as a type of epidural technique; however, we identified no relevant recent publications addressing the relationship between adult caudal anaesthesia or analgesia and StEP-advocated perioperative outcomes. A summary of contemporary articles identified by our search strategy and included in narrative discussion is provided in Table 2.

**Table 2:** Characteristics of included contemporary articles reporting neuraxial techniques and impact on standardised perioperative outcomes. CI, confidence interval; ERAS, enhanced recovery after surgery; HR, hazard ratio; IQR, inter-quartile range; NRS, numerical rating scale; OR, odds ratio; QoR-9; nine-point Quality of Recovery questionnaire; QoR-15; fifteen-point Quality of Recovery questionnaire; RD, risk difference; RR, relative risk; StEP, Standardised Endpoints in Perioperative Medicine. ∗Determined by institutional sponsorship for returned primary research or consensus guidelines, or institution of first or corresponding author for returned secondary research or opinion articles. ^†^Tabulated comparisons only include those relevant to this review, individual articles may contain additional comparator groups which are not included in this table. ^‡^Tabulated perioperative outcomes only include those found in StEP domains, individual articles may contain additional non-StEP advocated outcomes of potential interest but are not included in this table. Outcomes include both primary and secondary (exploratory) outcomes reported in primary research.

First author, year, country of origin∗	Design	Population	Comparison of relevance (intervention/control)^†^	Relevant StEP-advocated perioperative outcomes reported^‡^	Main conclusions relevant to StEP outcome domains
Koning, 2018, Netherlands	Single-centre randomised trial	Laparoscopic abdominal surgery	Spinal with intrathecal opioid/Sham spinal with i.v. opioid	Pain intensity, patient satisfaction, time to mobilisation, nausea and vomiting	Spinal with intrathecal opioid associated with lower pain in the first 24 postoperative hours (median [IQR] 11-point NRS: 0.3 [0–3.8] *vs* 2.3 [1.3–4.3]; *P*=0.004.
Salicath, 2018, UK	Systematic review meta-analysis	Abdominal surgery	Epidural analgesia/i.v. patient-controlled analgesia	Pain intensity, nausea and vomiting, thromboembolism, death	Epidural analgesia reduces pain intensity at rest (moderate quality evidence, small effect) and on movement (low quality evidence, moderate effect). No differences detected in other outcomes.
O'Donnell, 2018, UK	Systematic review meta-analysis	Hip fracture	Spinal anaesthesia/general anaesthesia	Acute renal failure, pneumonia, myocardial infarction, death	There was no significant difference in 30-day mortality (OR 1.04 [95% CI 0.99–1.10]), pneumonia (OR 1.03 [95% CI 0.91–1.17]), myocardial infarction (OR 0.96 [95% CI 0.87–1.05]) for spinal compared with general anaesthesia.
Gebhardt, 2018, Germany	Single-centre randomised trial	Knee arthroscopy	Spinal anaesthesia/general anaesthesia	Pain intensity, patient satisfaction, nausea and vomiting, quality of recovery	No significant group differences in overall postoperative satisfaction or quality of recovery on QoR-9.
Cummings, 2018, USA	Retrospective database analysis	Surgery for colorectal cancer	Epidural analgesia/non-epidural analgesia	Surgical site infection, sepsis, acute kidney injury, myocardial infarction, pneumonia, postoperative tracheal intubation, venous thromboembolism, prolonged mechanical ventilation, death	For open surgery: epidural analgesia was associated with fewer cardiorespiratory complications (OR 0.58 [95% CI 0.35–0.95]; *P*=0.03).
Kowark, 2018, Germany	Multicentre randomised trial	Age ≥65 yr with hip fracture	Spinal/general anaesthesia	Pain intensity, patient satisfaction, functional status, discharge disposition, death, non-fatal cardiac arrest, myocardial infarction, pneumonia, pulmonary embolism, unplanned postoperative tracheal intubation, prolonged mechanical ventilation	Published trial protocol: recruitment ongoing.
Brovman, 2019, USA	Retrospective database analysis	Ankle fracture fixation surgery	Neuraxial, and/or peripheral nerve block anaesthesia/general anaesthesia	Surgical site infection, urinary tract infection, sepsis, myocardial infarction, acute kidney injury, pneumonia, unplanned postoperative tracheal intubation, prolonged mechanical ventilation, venous thromboembolism, death	No significant differences detected in any outcomes of neuraxial and/or peripheral nerve block anaesthesia and general anaesthesia.
Ban, 2019, USA	Narrative review	Colorectal surgery	Neuraxial analgesia/control	Pain intensity, nausea and vomiting, morbidity	For open surgery: compared with opioids, epidural analgesia provides superior postoperative analgesia and decreases some cardiorespiratory morbidity.
Baar, 2019, Germany	Retrospective database analysis	Living donor nephrectomy	Epidural analgesia/non-epidural analgesia	Renal replacement therapy	Epidural analgesia associated with reduced delayed graft function.
Wink, 2019, Netherlands	Narrative review.	Adult perioperative practice	Epidural analgesia and anaesthesia	Myocardial infarction	*‘A potential influence of thoracic epidural anaesthesia on the incidence of perioperative myocardial infarction is favoured by some studies but remains to be clarified.’*
Vester-Andersen, 2020, Denmark	Retrospective database analysis	Emergency abdominal surgery	Epidural analgesia/non-epidural analgesia	Death	Epidural analgesia associated with decreased risk of mortality in adjusted analysis at 30 days (OR 0.75 [95% CI 0.62–0.90]; *P*<0.01) and 90 days (OR 0.80 [95% CI 0.67–0.94]; *P*=0.01).
Malhas, 2019, Canada	Retrospective database analysis	Hip fracture	Spinal anaesthesia/general anaesthesia	Major adverse cardiac events, pulmonary embolism, death	Spinal anaesthesia associated with lower risk of pulmonary embolism (RR 0.36 [95% CI 0.12–0.60]; *P*<0.001), and 90-day mortality (RR 0.74 [95% CI 0.52–0.96]; *P*=0.037) but not major adverse cardiac events or death at 30 or 60 days.
Kaufmann, 2019, Germany	Retrospective database analysis	Oesophagectomy	Epidural analgesia/non-epidural analgesia	Postoperative pulmonary complications, unplanned postoperative tracheal intubation, death	Absence of epidural analgesia associated with postoperative pulmonary complications (OR 2.0 [95% CI 1.01–3.8]) and death (OR 3.9 [95% CI 1.6–9.7]).
Tang, 2020, Australia	Retrospective database analysis	Open liver resection	Spinal with intrathecal opioid/multimodal analgesia alone	Pain intensity, nausea and vomiting, morbidity	Intrathecal opioid led to significantly reduced postoperative pain intensity in the first 24 postoperative hours (median [IQR] 11-point NRS 3 [1–5] *vs* 4 [3–6]; *P*=0.007).
Desai, 2021, UK	Systematic review meta-analysis	Abdominal surgery	Epidural/transversus abdominis plane (TAP) block	Pain intensity, nausea and vomiting, surgical site infection, time to mobilisation, quality of recovery	Epidural analgesia statistically superior to TAP block in 12-h pain intensity at rest, but this difference is not clinically important.
Roberts, 2020, Canada	Retrospective database analysis	Lower limb revascularisation surgery	Neuraxial/general anaesthesia	Major adverse cardiac events, pneumonia, venous thromboembolism, acute kidney injury, death	Neuraxial anaesthesia associated with decreased mortality (adjusted OR 0.72 [95% CI 0.58–0.89]; *P*<0.001) and major adverse cardiac events (adjusted OR 0.72 [95% CI 0.60–0.87]; *P*<0.001).
Liu, 2020, China	Single-centre randomised trial	Age ≥65 yr undergoing laparoscopic colorectal surgery	Epidural with general anaesthesia/general anaesthesia alone	Pain intensity, nausea and vomiting, quality of recovery	Mean (standard deviation) QoR-15 at 24 h significantly higher following epidural analgesia (110.6 [4.4] *vs* 100.6 [6.5]; *P*<0.001).
Johnson, 2020, USA	Retrospective database analysis	Age ≥50 yr undergoing lower limb joint arthroplasty	Neuraxial anaesthesia/general anaesthesia	Death	Among frailty-vulnerable patients, neuraxial anaesthesia was associated with improved survival (HR 0.49 [95% CI 0.27–0.89]). No difference in risk between anaesthetic technique was observed in frail or non-frail cohorts.
Howle, 2022, Ireland	Systematic review network meta-analysis	Midline laparotomy	Neuraxial analgesia/abdominal wall blocks/wound infiltration/control	Pain intensity, nausea and vomiting, time to mobilisation	Low-quality evidence that epidural analgesia is associated with clinically superior pain scores compared with alternative continuous regional anaesthesia techniques for the first 24 postoperative hours
Kendall, 2021, USA	Retrospective database analysis	Primary total knee arthroplasty	Spinal/general anaesthesia	Surgical site infection, sepsis, renal failure, renal insufficiency, myocardial infarction, cardiac arrest, thromboembolic event, pneumonia, unplanned postoperative intubation, prolonged mechanical ventilation, death	General anaesthesia associated with higher incidence of minor adverse events (2.09% *vs* 0.51%; *P*<0.001) but not difference in serious adverse events (0.92% *vs* 0.66%; *P*=0.369) compared with spinal anaesthesia.
Dieu, 2021, Switzerland	Consensus recommendation	Open liver resection	Epidural analgesia/spinal intrathecal opioid/non-neuraxial analgesia	Pain intensity	Epidural analgesia, or bilateral subcostal TAP blocks, are recommended.
Du, 2021, China	Multicentre randomised trial	Age ≥60 yr undergoing major thoracic or abdominal surgery	Epidural analgesia/i.v. analgesia	Cancer-specific survival, recurrence-free survival, event-free survival, quality of life, overall survival	Epidural analgesia had no effect on overall, cancer-specific, recurrence-free, or event-free survival.
Wang, 2021, China	Systematic review meta-analysis	Primary total hip or knee arthroplasty	Spinal with intrathecal morphine/spinal without intrathecal morphine	Nausea and vomiting	No difference in incidence of nausea and vomiting (RR 1.21 [95% CI 0.98–1.49]; *P*=0.08).
Neuman, 2021, USA	Multicentre randomised trial	Age ≥50 yr with hip fracture	Spinal/general anaesthesia	Death, functional status, myocardial infarction, non-fatal cardiac arrest, pneumonia, pulmonary embolism, unplanned postoperative intubation, surgical site infection, acute kidney injury, urinary tract infection, time to mobilisation, discharge disposition	Spinal anaesthesia was not superior to general anaesthesia with respect to survival and recovery of ambulation at 60 days (RR 1.03 [95% CI 0.84–1.27]; *P*=0.83).
Li, 2022, China	Multicentre randomised trial.	Age ≥65 yr with hip fracture	Neuraxial with no sedation/general anaesthesia	Death, nausea and vomiting, myocardial infarction, pneumonia, stroke	No difference in all-cause mortality at 30 days between neuraxial and general anaesthesia (RR 2.0 [95% CI 0.0.6–6.5]).
Falk, 2021, Sweden	Multicentre randomised trial	Surgery for colorectal cancer	Epidural analgesia/patient-controlled i.v. opioid analgesia (PCA)	Disease-free survival, pain intensity, morbidity	Significantly lower pain intensity on activity with epidural *vs* PCA on the first postoperative day. No significant difference between the epidural and PCA in disease-free survival (adjusted HR 1.19 [95% CI 0.61–2.31]; *P*=0.61).
Xu, 2021, China	Multicentre randomised trial	Video-assisted thoracoscopic surgery for lung cancer	Epidural anaesthesia–analgesia/general anaesthesia alone	Recurrence-free survival, overall survival, cancer-specific survival	Epidural analgesia had no effect on overall, cancer-specific, recurrence-free survival compared with general anaesthesia alone.
Pirie, 2022, Australia	Narrative review	Major abdominal surgery	N/A	Pain intensity, quality of life, patient satisfaction, time to mobilisation, nausea and vomiting, morbidity, death	*‘Limited research exists on patient quality of recovery using specific analgesic techniques after intra-abdominal surgery. Poorly controlled postoperative pain after major abdominal surgery should be a research priority’.*
El-Boghdadly, 2022	Systematic review with qualitative synthesis	Colorectal surgery with ERAS protocol	Regional anaesthesia or analgesia/no, or placebo, regional anaesthesia or analgesia	Pain intensity, nausea and vomiting, time to mobilisation, quality of recovery, death	*‘Epidural analgesia had limited evidence of outcome benefits in open surgery, while spinal analgesia with intrathecal opioids may potentially be associated with improved outcomes with no impact on length of stay in laparoscopic surgery, though dosing must be further investigated’.*
Feray, 2022, Switzerland	Consensus recommendation	Video-assisted thoracoscopic surgery	Neuraxial analgesia/non-neuraxial analgesia	Pain intensity	Epidural analgesia not recommended for postoperative analgesia in video-assisted thoracoscopy
Okuda, 2022, Japan	Single-centre randomised trial	Video-assisted thoracoscopic surgery	Epidural analgesia/general anaesthesia alone	Pain intensity, respiratory failure, pneumonia	No differences detected between groups in these secondary outcomes.
Hasselager, 2022, Denmark	Retrospective database analysis	Surgery for colorectal cancer	Epidural analgesia/general anaesthesia	Cancer recurrence, death	No association between epidural analgesia and recurrence (HR 0.91 [95% CI 0.82–1.02]) or mortality (HR 1.01 [95% CI 0.92–1.10]).
Kunutsor, 2022, UK	Systematic review meta-analysis	Hip fracture	Spinal anaesthesia/general anaesthesia	Pain intensity, quality of life, acute kidney injury, pneumonia, acute coronary syndrome, death	Spinal anaesthesia reduced the risk of acute kidney injury (RR 0.59 [95% CI 0.39–0.89]). There were no significant differences in other outcomes.
Lin, 2023, Australia	Retrospective database analysis	Hip fracture	Spinal anaesthesia/general anaesthesia	Death	Spinal anaesthesia not associated with altered risk of long-term death compared with general anaesthesia (adjusted HR 1.03 [95% CI 0.96–1.11]). Combined spinal and general anaesthesia associated with increased risk of long-term death (adjusted HR 1.12 [95% CI 1.02–1.24]).

## Discussion

### Patient comfort and patient-centred outcomes

#### Orthopaedic and ambulatory surgery

Large, retrospective studies and consensus reviews published since 2018 have established the role of neuraxial anaesthesia for lower limb joint arthroplasty.^14^^,^^15^ Reported patient-centred benefits of neuraxial anaesthesia compared with general anaesthesia include decreased length of hospital stay, postoperative pain, opioid consumption, and incidence of postoperative nausea and vomiting (PONV). Two systematic review and meta-analyses (SRMAs) published in the preceding 5 years have reported significant analgesic benefit of intrathecal opioid for arthroplasty surgery, but at the reported cost of higher rates of nausea and vomiting^16^ and pruritis.^17^ Both reviews conclude that additional research is required to explore optimal intrathecal opioid dosing. High-quality evidence reported in 2021 demonstrates that intrathecal morphine (in common with fentanyl and diamorphine) is no more likely to cause respiratory depression than controls who received no intrathecal opioid.^16^

Several recent large RCTs have attempted to determine whether neuraxial anaesthesia has benefits compared with general anaesthesia for hip fracture surgery.^18^, ^19^, ^20^ One of the most prominent trials, Regional versus General Anesthesia for Promoting Independence after Hip Fracture (REGAIN), included patient-centred outcomes such as the inability to walk without human assistance at 60 days, discharge disposition, and worsened walking ability.^18^ There was no difference between spinal and general anaesthesia in the inability to walk independently at 60 days (15.2% and 14.4%, respectively; relative risk [RR] 1.05, 95% confidence interval [CI] 0.82–1.36), discharge disposition (home, long-term care facility, hospice, rehabilitation), or worsened walking ability. The pragmatic design of REGAIN (e.g. purposefully broad eligibility criteria and the non-protocolisation of intervention and control arm treatments) replicates ‘real-world’ practice, but nevertheless clinical circumstances (e.g. the presence of active respiratory infection, or the skill of an individual anaesthetic practitioner in a particular technique) may mean one anaesthetic method is preferred over another in specific circumstances.

The advent of fast-track general anaesthesia techniques, which emphasise avoidance of benzodiazepines and longer-acting opioids, has challenged the previously held theory that spinal anaesthesia provides a superior recovery profile for ambulatory surgery patients. Perceived shortcomings of spinal anaesthesia include postoperative urinary retention, risk of transient neurologic symptoms (TNS), and delayed recovery of motor function, particularly with longer-acting local anaesthetics. The resurgence of chloroprocaine as an intrathecal anaesthetic agent has mitigated some of these concerns, because it is short-acting with a low incidence of urinary retention and TNS.^21^ A recent RCT comparing propofol-based general anaesthesia with spinal anaesthesia (40 mg chloroprocaine) with propofol-based sedation in patients undergoing outpatient knee arthroscopy demonstrated a faster time to discharge, longer time to first pain, and decreased cost with spinal anaesthesia.^22^ There was no difference between groups in terms of incidence of PONV, QoR-9 score, or patient satisfaction. Another large, multicentre observational study in ambulatory patients undergoing mostly urological and orthopaedic surgery found that general anaesthesia demonstrated shorter times to urination and ambulation compared with spinal anaesthesia, although there was no difference in time to discharge.^23^

#### Abdominal surgery

Quality of life measurement reporting in the literature is rare, as even recent studies continue to focus on the endpoints of pain, opioid consumption, and adverse events. Thoracic epidural analgesia (TEA) had previously been described as the gold standard for reducing postoperative pain and respiratory complications after major abdominal surgery.^24^ Recently, a preference for minimally invasive surgical approaches has led to a decline in the use of epidural analgesia and a transition to intrathecal opioids and truncal nerve/plane blocks.^25^ The use of spinal analgesia with intrathecal opioids is associated with improved pain outcomes compared with i.v. opioids alone in laparoscopic^26^ and open^27^ abdominal surgery, although opioid dosing remains an area of uncertainty.^28^

There remains a role for TEA in open colorectal and hepatic surgeries. One review found that TEA was associated with superior pain control when compared with non-TEA analgesic protocols in open colorectal surgery.^29^ Similarly, 2019 Enhanced Recovery After Surgery (ERAS®) Society Guidelines for Perioperative Care in Elective Colorectal Surgery recommend TEA as part of a multimodal analgesic protocol in open surgeries.^30^ These recommendations are made despite the known higher incidence of major (permanent nerve injury) and minor (hypotension and urinary retention) adverse events with TEA compared with spinal or truncal nerve block.^31^, ^32^, ^33^

TEA is not generally recommended in laparoscopic surgeries, especially when considering traditional outcomes such as pain, opioid consumption, and adverse events including hypotension and urinary retention.^25^^,^^29^^,^^30^ There may, however, be benefits to TEA in laparoscopic surgery for certain patient subgroups. Liu and colleagues^34^ examined the effects of intraoperative TEA alongside general anaesthesia compared with general anaesthesia alone in the quality of recovery of older adults undergoing laparoscopic radical colonic resection for cancer. Patients with TEA had improved QoR-15 scores compared with general anaesthesia alone (110.60 *vs* 100.63, *P*<0.001 at 24 h; 116.43 *vs* 112.63, *P*=0.006 at 72 h).

Fascial plane blocks are popular components of modern multimodal analgesia after abdominal surgery. Since 2018, there have been several meta-analyses comparing the effect of transversus abdominis plane (TAP) blocks and TEA on outcomes after abdominal surgery. One SRMA examining 1220 patients in 18 RCTs, 13 of which involved open surgery, demonstrated a decrease in opioid consumption and pain scores at rest and on movement at 12 and 48 h in the TEA group, although these differences did not meet the prespecified threshold for clinical significance.^35^ The quality of evidence contributing to the SRMA did not allow conclusions regarding other patient-centred outcomes such as PONV, recovery of intestinal function, or quality of recovery. Another SRMA, examining 568 patients undergoing mostly laparoscopic abdominal surgery in six RCTs, found no analgesic difference between TAP and TEA with the exception of improved pain with movement at 24 h, where TEA was deemed statistically, but not clinically, superior.^36^ TAP block was determined to be superior to TEA with respect to opioid consumption, time to ambulation, and duration of urinary catheterisation. This SRMA lends support to the use of TAP block over TEA for laparoscopic surgery. In contrast, for midline laparotomy, an SRMA and separate network meta-analysis including 36 trials with 2056 subjects indicated TEA to be superior to truncal facial plane block in pain scores and opioid consumption at 24 h.^37^

There is a benefit using TEA in open hepatic surgery, with TEA demonstrating lower pain scores than alternate analgesic protocols, including wound catheters.^25^ The Procedure-Specific Postoperative Pain Management (PROSPECT) working group examined 31 RCTs and three systemic reviews in order to develop recommendations for pain management after open liver resection, published in 2021.^38^ The group determined that TEA provided better pain control in the first 24 h compared with subcostal TAP blocks and catheters, and also performed better than paravertebral blocks (PVBs). The PROSPECT recommendations for open hepatic surgery included paracetamol, NSAIDs, and either TEA or subcostal TAP blocks after surgery.

#### Thoracic surgery

Thoracic surgery is increasingly performed using less invasive, video-assisted thoracoscopic (VAT) techniques.^39^^,^^40^ The PROSPECT working group reviewed RCTs published during 2010–21 to develop guidelines for postoperative pain control in people undergoing VAT procedures, published in 2022.^41^ The group strongly recommended the inclusion of a regional analgesic technique to a standard multimodal protocol, with a preference for PVB over TEA. Although studies showed that TEA provided better or equivalent pain control to PVB, TEA is associated with a greater incidence of significant side-effects.^42^

As with abdominal surgery, studies examining other patient-centred outcomes beyond pain control in thoracic surgery are few. One recent, albeit small, study examined health-related quality of life (HRQoL) in 65 patients undergoing thoracoscopic-laparoscopic oesophagectomy randomised to either intraoperative combined TEA and general anaesthesia or general anaesthesia alone.^43^ All patients then received postoperative pain relief with TEA. European Organisation for Research and Treatment of Cancer (EORTC) Quality of Life scores were assessed before surgery and at 7 and 30 days after surgery. The combined TEA–general group had better HRQoL scores, particularly in the social, emotional, and global health domains. Symptoms of sleep disorders, nausea, constipation, reflux, and cough difficulty were also less severe in the combined TEA–general group. Pain was also less severe in the combined group, although this finding was considered not to be clinically significant.

### Infection and sepsis

Taken together with pulmonary and urinary tract infections (addressed elsewhere in this review), surgical site infections (SSIs) account for the majority of postoperative infections.^44^ Given that SSIs complicate 0.5–3% of surgeries,^45^ result in an average prolongation of hospital stay by 7–11 days,^46^^,^^47^ and a 78% readmission rate in patients discharged home,^48^ testing of interventions to reduce SSI incidence, severity, or both has been a major research priority. Among known modifiable perioperative risk factors, prevention of sympathetic catecholamine-induced vasoconstriction and improved tissue oxygenation have been offered as potential mechanisms by which neuraxial anaesthesia may positively impact on acute tissue healing and therefore SSI rates.^49^, ^50^, ^51^, ^52^, ^53^ The relative preservation of immune function arising from the decreased surgical stress response, reduced allogenic blood transfusion rates, and improved pain control with associated reduced sympathetic activation, all observed with neuraxial anaesthesia and analgesia, may also contribute to a reduced incidence of subsequent SSI.^54^, ^55^, ^56^, ^57^, ^58^

The potentially catastrophic consequences of SSI after joint arthroplasty^59^^,^^60^ and the fact that lower limb surgery can be performed using either neuraxial or general anaesthetic means this surgical group has been intensively studied with regard to SSI and anaesthetic technique. In a landmark population study published in 2010, Chang and colleagues^61^ reported an adjusted odds ratio (aOR) of 2.21 (95% CI 1.25–3.90) for SSI under general anaesthesia *vs* neuraxial anaesthesia among 3081 patients who underwent arthroplasty surgery. Further retrospective analyses of registry data yielded conflicting results. One large observational study of 16 555 patients supported a positive impact of neuraxial anaesthesia on overall infection rate after joint arthroplasty; however, this effect was not observed for SSIs specifically.^62^ Similarly, analysis of 56 216 patients undergoing total knee arthroplasty at 45 hospitals reported no significant effect of anaesthetic type on rates of deep SSI.^63^ In the absence of RCTs conducted on this topic, meta-analysis of published observational data is likely to provide the most compelling evidence, acknowledging the unavoidable methodological weaknesses of non-experimentally derived data. Analysing a total of 362 029 patients undergoing lower limb arthroplasty, Zorrilla-Vaca and colleagues^64^ reported neuraxial anaesthesia to be associated with a significant reduction in SSI compared with general anaesthesia (aOR 0.84, 95% CI 0.76–0.92). Addressing all forms of postoperative infection (rather than limited to SSI), further support for neuraxial techniques was reported by Memtsoudis and colleagues'^65^ analysis of 382 236 cases of arthroplasty, and, most recently, for all forms of regional anaesthesia by Wan and colleagues'^44^ analysis of 39 996 subjects prospectively gathered cases across multiple surgical specialties (OR 0.78, 95% CI 0.69–9.87). Of note, all these studies were reported before the most recent 5-year period. We were unable to identify more contemporary published evidence to further inform this topic.

In summary, whether neuraxial anaesthesia has an impact on SSI after surgery has been most extensively investigated after elective orthopaedic surgery. There is evidence that neuraxial anaesthesia reduces the risk of SSI compared with general anaesthesia; however, the certainty of this evidence is significantly weakened by underlying issues of internal validity arising from confounding and selection bias in observational research. In comparison with evidence supporting other infection-reducing modifications to the perioperative pathway, the likely contribution of neuraxial *vs* general anaesthetic technique to SSI is much less significant.

### Postoperative cancer outcomes

The exploration of links between neuraxial (and, more generally, regional anaesthetic) techniques and cancer outcomes provides an instructive example of the challenges of bench-to-bedside research addressing specific health needs. Enthusiasm, born of encouraging immunological data and animal models of disease, supported by promising retrospective analyses, has been tempered by the more recent publication of experimental clinical trials demonstrating no link between regional anaesthesia and cancer outcomes in patients undergoing various forms of cancer resection surgery.

By reducing the neuroendocrine response to surgery, maintaining natural killer cell activity, and reducing the burden of circulating tumour cells, neuraxial anaesthesia offers plausible cellular benefit over general anaesthesia achieved with volatile agents and systemic opioids.^66^, ^67^, ^68^, ^69^, ^70^, ^71^, ^72^ Neuraxial anaesthesia may, therefore, offer a cancer benefit via an opioid-sparing effect, volatile-sparing effect, or both. In murine models of natural killer cell activity and cancer metastases under general anaesthesia, the addition of spinal anaesthesia significantly attenuated perioperative metastatic burden for breast^73^ and liver^74^ cancer. Identifying clinically important benefit from these cellular and animal findings has, however, proved elusive. Observational interrogation of procedural databases demonstrated possible increase in time to tumour recurrence and 3- and 5-year overall survival in patients with ovarian cancer,^75^^,^^76^ reduced risk of biochemical (i.e. prostate-specific antigen) recurrence and clinical cancer progression after radical prostatectomy,^77^^,^^78^ decreased all-cause mortality in patients with rectal cancer,^79^ and improved recurrence and metastasis-free survival in patients after surgery for breast cancer.^80^ Despite several retrospective and *post hoc* secondary analyses (and subsequent meta-analysis) showing no significant improvements in recurrence rates, disease-free survival, or overall survival with neuraxial anaesthesia,^81^, ^82^, ^83^, ^84^, ^85^, ^86^, ^87^ the question of whether fundamental choices in analgesic technique could affect time to tumour progression, disease-free survival, cancer-specific survival, or overall survival (all recommended core outcome measures, see Table 1) is clearly deserving of robust prospective experimental assessment.

Such assessment has been provided in several recently published studies. In a pre-planned, 5-year follow-up of 1712 older adults randomised to either combined epidural–general anaesthesia or general anaesthesia alone for major thoracoabdominal surgery, there was no difference in overall (adjusted hazard ratio [aHR] 1.07, 95% CI 0.92–1.24), cancer-specific (aHR 1.09, 95% CI 0.93–1.28), recurrence-free (aHR 0.97, 95% CI 0.84–1.12), or event-free survival (aHR 0.99, 95% CI 0.86–1.12).^88^ Epidural analgesia did not, therefore, improve overall or cancer-specific long-term mortality in study participants, even though exposure to volatile and opioid medications was reduced in the epidural group. In a further RCT of 400 patients undergoing VAT surgery for lung cancer, combined epidural–general anaesthesia was no different to general anaesthesia in terms of recurrence-free (aHR 0.90, 95% CI 0.60–1.35), overall (aHR 1.12, 95% CI 0.64–1.96), or cancer-specific survival (aHR 1.08, 95% CI 0.61–1.91).^89^ Although, in both trials, some patients underwent surgery for non-cancer disease (8% and 16%, respectively), the groups were balanced in this regard. In an RCT of 221 patients undergoing colorectal cancer surgery, disease-free survival was not improved by the provision of epidural analgesia in place of i.v. morphine (aHR 1.19, 95% CI 0.61–2.31).^90^ Importantly, the above trials enrolled patients undergoing neurohumoral stress-provoking surgery and cannot be subject to the same analysis as trials assessing the impact of PVB in breast cancer surgery,^91^^,^^92^ namely that negative trial findings arose because the surgery itself elicits an insufficient host stress response.

In conclusion, *in vitro* and animal studies notwithstanding, recent observational and experimental human data indicate that neuraxial techniques have no significant effect on cancer recurrence or survival and that clinicians and patients should make decisions on whether to deploy neuraxial techniques based on other intended outcomes and patient preference.

### Renal endpoints

The acute kidney injury (AKI) syndrome is described as a ‘*sentinel postoperative complication*’^93^ because, in addition to direct patient harm, it is an independent contributor to chronic kidney disease, concurrent extra-renal complications, prolonged critical care stay, length of hospital stay, increased healthcare costs, and death.^94^, ^95^, ^96^, ^97^, ^98^, ^99^, ^100^, ^101^ Observed in 6.1–13.4% of patients undergoing major noncardiac surgery,^97^^,^^102^ AKI exists on a continuum with acute kidney disease and chronic kidney injury.^103^^,^^104^ In addition to AKI, StEP-advocated renal outcome measures are acute kidney disease, composite of death or renal replacement therapy, and a composite of major adverse kidney events (Table 1).

Most cases of postoperative AKI are acknowledged to have a multifactorial aetiology, with preoperative (e.g. diabetes mellitus, pre-existing kidney dysfunction), intraoperative (e.g. systemic and renal hypotension),^105^, ^106^, ^107^ and postoperative (e.g. nephrotoxin exposure) risk factors contributing to a common injury pathway of disrupted microcirculation, tissue inflammation, and parenchymal ischaemia.^93^ Given this multifactorial aetiology, it would appear unlikely that isolated single modifications to modern intraoperative anaesthetic technique (the choice of general *vs* neuraxial anaesthesia, for example) will, in itself, deliver significant improvement in the incidence or severity of postoperative renal outcomes.^108^ Perhaps because of this, in contrast with outcomes related to patient comfort, comparatively few recent prospective studies of neuraxial *vs* general anaesthesia in noncardiac surgery address renal outcomes as their primary outcome.

Nevertheless, biologically plausible explanations can be offered to support possible effects of neuraxial anaesthesia on the kidney. The sympathetic nerve supply to the kidneys (T7–T11) decreases renal blood flow and increases tubular sodium resorption and renin secretion.^109^ Given that the sympathetic nervous system is commonly disturbed by neuraxial techniques, some effect on acute renal physiology (if not long-term renal outcome) could reasonably be expected. Putative beneficial effects of neuraxial anaesthesia include altered microcirculation,^110^ reduced stress hormone response to surgery,^111^ and improved postoperative coagulation.^112^ The extent to which technique itself, rather than perioperative confounders such as concomitant fluid regimes and exposure to intraoperative hypotension, contribute to renal outcomes is unknown, and the direct comparative effects of neuraxial *vs* general anaesthesia are also uncertain. Although there is a no significant decrease in renal blood flow in healthy volunteers undergoing mid-thoracic neuraxial block,^113^ whether this is the case in patients at risk of adverse renal outcomes (e.g. patients with diabetes mellitus or chronic renal vascular disease) is not clear.

Turning to recent literature addressing outcomes, a retrospective population-data analysis of patients undergoing lower limb revascularisation surgery (and therefore at substantial risk of AKI) demonstrated that patients undergoing neuraxial anaesthesia had an unadjusted OR of AKI of 0.48 (95% CI 0.34–0.67) compared with patients receiving general anaesthesia.^114^ Owing to lack of statistical power, the authors could only perform regression adjustment for confounders on a composite outcome combining cardiac, renal, and pulmonary complications, so the extent of any true effect on renal outcome by anaesthetic technique remains uncertain. Similarly, in patients undergoing lower limb arthroplasty, meta-analysis has demonstrated that neuraxial anaesthesia is protective against acute postoperative renal failure, especially when administered as a sole technique, rather than in addition to general anaesthesia (OR 0.69, 95% CI 0.59–0.81, *P*<0.0001 for total hip arthroplasty; OR 0.73, 95% CI 0.65–0.82, *P*<0.0001 for total knee arthroplasty).^14^ In the special circumstance of living kidney donation, the addition of epidural analgesia to general anaesthesia has been retrospectively linked with a lower rate of delayed graft function,^110^ but further prospective data are needed to validate this hypothesis-generating, retrospective study.

### Cardiovascular outcomes

The readily observable acute alterations in cardiovascular status, including heart rate, arterial blood pressure, and vasomotor tone induced by neuraxial techniques,^115^^,^^116^ and experimental evidence that TEA improves coronary function and myocardial oxygen balance in ischaemic heart disease^117^, ^118^, ^119^, ^120^, ^121^ has led researchers to continue to examine whether neuraxial analgesia could positively impact perioperative endpoints of myocardial health in surgical populations. There is conflicting *post hoc* observational^122^ and experimental^123^^,^^124^ evidence regarding the association between neuraxial anaesthesia (either as a sole technique or in combination with general anaesthesia) and myocardial infarction or various composite outcomes encompassing cardiovascular or cardiopulmonary morbidity after noncardiac surgery. In the decade preceding our literature search, two highly cited meta-analyses showed that TEA did not demonstrate a reduced risk of myocardial infarction.^125^^,^^126^ Pöpping and colleagues^125^ analysed data from 9044 patients in 125 trials encompassing all types of surgery up to 2012. They identified a non-statistically significant effect of epidural analgesia in reducing the rate of myocardial infarction compared with non-epidural regimes (OR 0.73, 95% CI 0.50–1.06), but did detect significantly reduced rates of atrial fibrillation (OR 0.63, 95% CI 0.49–0.82) and supraventricular tachycardia (OR 0.69, 95% CI 0.55–0.87). Similarly, a 2014 meta-analysis of data from 849 subjects undergoing any form of open or laparoscopic surgery reported no difference in risk of myocardial infarction between neuraxial and general anaesthetic techniques (RR 1.17, 95% CI 0.57–2.37) or between combined neuraxial–general anaesthesia *vs* general anaesthesia alone (RR 0.69, 95% CI 0.44–1.09). This contrasts with a meta-analysis of 1498 patients undergoing exclusively open aortic surgery which showed that adding an epidural to general anaesthesia reduced the rate of myocardial infarction (RR 0.54, 95% CI 0.30–0.97; number needed to treat [NNT] for one additional beneficial outcome 28).^127^ The differential effect of epidural analgesia on cardiac outcome appears to depend on the degree of systemic surgical insult. The physiological challenge of open thoracoabdominal surgery may offer neuraxial block the opportunity to prevent cardiovascular harm that laparoscopic surgeries do not. This was demonstrated in a retrospective, propensity-matched analysis of patients undergoing colorectal surgery using the American College of Surgeons National Surgical Quality Improvement Program (NSQIP) data.^128^ Although the 1611 patients who underwent open colectomy with TEA experienced fewer cardiopulmonary complications than matched patients without epidural (OR 0.58, 95% CI 0.35–0.95), this effect was not observed when both open and laparoscopic surgeries were analysed (OR 0.87, 95% CI 0.68–1.11).

In conclusion, current evidence indicates that TEA exerts a beneficial effect on cardiovascular outcomes in specific pathologies such as medically resistant angina pectoralis and for surgical populations undergoing major open procedures in terms of myocardial infarction. Translating population-level data on cardiovascular benefit to inform the care of individual patients undergoing specific surgery is challenging. Potential protection from perioperative ischaemic heart disease may be offset by the neuraxial sympathectomy causing greater perioperative haemodynamic instability, especially in high-risk patients,^116^ with higher rates of perioperative hypotension being a consistent finding in clinical trials.^129^

### Pulmonary complications

Reducing the risk of postoperative pneumonia, respiratory failure, and mechanical ventilation continues to be one of the major justifications offered by clinicians for TEA in major thoracoabdominal surgery, even though the baseline risk of such events has decreased markedly over the past 40 years, probably owing to advances in surgical technique, perioperative monitoring, on-demand analgesia systems, and protocolisation of enhanced recovery after surgery.^130^ The association between effective analgesia and the ability to engage in postoperative respiratory therapy after major thoracoabdominal surgery is readily observable in clinical practice, and the natural history and pathophysiology of postoperative pulmonary complications is well described.^131^ Even so, it is striking that even in high-risk surgeries such as oesophagectomy via thoracotomy, the evidence that TEA reduces the incidence of pulmonary complications is inconsistent.^132^, ^133^, ^134^, ^135^, ^136^, ^137^ Across all forms of surgery, the risk of respiratory depression (OR 0.61, 95% CI 0.39–0.93), atelectasis (OR 0.67, 95% CI 0.48–0.93), and pneumonia (OR 0.56, 95% CI 0.45–0.70) is reduced by epidural analgesia. An RR reduction of 25% in total composite postoperative pulmonary outcomes was reported by Odor and colleagues^138^ in their 2020 meta-analysis of data derived from all noncardiac surgeries. A 2016 meta-analysis demonstrating a reduced rate of postoperative respiratory failure in patients undergoing open abdominal aortic aneurysm repair (OR 0.69, 95% CI 0.56–0.85) informed the 2022 ERAS Society and Society for Vascular Surgery consensus statement on open aortic perioperative care that ‘*Mid-thoracic (T6-T9) epidural analgesia is recommended intraoperatively*’.^139^

Importantly, the observed beneficial effect of TEA on pneumonia appears to have decreased over time, with one analysis showing a historical NNT of 4 using data from the 1970s but NNT of 25 using trial data to 2015 when TEA is compared with non-TEA control analgesia. The historical bias favouring epidural and other neuraxial techniques in comparison with non-neuraxial analgesia was acknowledged in multidisciplinary pain guidance endorsed by the American Pain Society, American Society of Regional Anesthesia and Pain Medicine, and the American Society of Anesthesiologists' Committee on Regional Anesthesia, which nevertheless stated clinicians should ‘*routinely consider use of epidural or spinal analgesia for management of postoperative pain in patients who undergo major thoracic and abdominal procedures, cesarean section, and hip and lower extremity surgeries, particularly in patients at risk for cardiac complications, pulmonary complications, or prolonged ileus*’.^140^

### Mortality

In the 5-year period covered by this literature search, we identified highly cited studies evaluating the impact of neuraxial techniques on mortality after emergency abdominal,^141^ elective arthroplasty,^14^ and hip fracture surgery.^18^ In their 2020 population-based cohort study enrolling 4920 adults undergoing emergency abdominal surgery, Vester-Andersen and colleagues^141^ reported an adjusted association between TEA and reduced 30-day (OR 0.75, 95% CI 0.62–0.90) and 90-day (OR 0.80, 95% CI 0.67–0.94) mortality. The authors theorise that the mortality benefit was observed because epidural analgesia reduces postoperative ileus and improves postoperative pain, mobilisation, deep breathing, and coughing, thereby reducing pulmonary complications and mortality. These effects may be most frequently observed in patients undergoing emergency laparotomy. This mortality benefit is consistent with previous population-based evidence^142^ and a meta-analysis of TEA in all forms of surgery conducted under general anaesthesia which showed a mortality rate of 2.0% among recipients of TEA *vs* 3.2% with non-TEA opioid-based analgesia (OR 0.69, 95% CI 0.51–0.92; NNT 90, 95% CI 55–244).^125^

In the setting of lower limb orthopaedic surgery, a 2019 meta-analysis of randomised and observation evidence by the International Consensus on Anaesthesia-Related Outcomes after Surgery (ICAROS) group demonstrated that neuraxial anaesthesia offered superior mortality compared with general anaesthesia for patients undergoing primary hip (OR 0.67, 95% CI 0.57–0.80) but not knee (OR 0.83 95% CI 0.60–1.15) arthroplasty.^14^ There is some evidence that the observed mortality benefit of neuraxial anaesthesia in arthroplasty may be particularly pertinent to patients vulnerable to frailty.^143^

In keeping with previously published retrospective data showing no difference between neuraxial and general anaesthesia on survival after hip fracture surgery,^144^, ^145^, ^146^ neither the REGAIN^18^ nor Effect of Regional *vs* General Anesthesia on Incidence of Postoperative Delirium in Older Patients Undergoing Hip Fracture Surgery (RAGA)^19^ trials found neuraxial anaesthesia to be superior to general anaesthesia in 60- or 30-day mortality, respectively. Meta-analysis of data from these and other trials, published in 2022, reported no difference in mortality up to 120 days between anaesthetic techniques,^147^ a finding in keeping with previous meta-analysis of both randomised and observational evidence showing no mortality benefit with neuraxial anaesthesia in hip fracture surgery.^148^

### Conclusions

In this review, we have synthesised pivotal research on neuraxial techniques published in the preceding 5 years relevant to the StEP initiative domains in patient comfort, infection, cancer, renal, cardiovascular, pulmonary, and mortality outcomes. In addition to these contemporary studies, we have explored returned citation lists to highlight the foundational scholarship upon which ongoing research into the relationship between patient outcome and epidural or spinal anaesthesia is based. It should be noted that several of the cited publications were published before the StEP initiative, and consequently, the fit of reported outcomes to those presented in this manuscript is imperfect. Nevertheless, we consider the use of the StEP framework to be important and useful. A consistent finding of this review, notable across all StEP domains, is that biological plausibility, mechanistic support, and observational association between neuraxial techniques and clinical outcomes is frequently reported in the literature, but convincing experimental evidence of causation between independent (anaesthetic technique) and dependent (patient outcome) variables in large clinical trials or meta-analysis is much less frequently observed. It is noteworthy that of the 34 articles returned by our search of the most recent 5 years of literature, 24 are secondary analyses and only 10 are clinical studies reporting new primary data.

This review has several limitations. We have not included environmental sustainability, cognition, or equality of access to treatment as outcome domains of interest because these do not feature as current StEP domains; however, we acknowledge that these are of increasing professional concern and are likely to feature heavily in future studies examining neuraxial techniques. Our intention in performing this review was to provide a narrative description of key recent outputs on this topic, based on a structured search of the published literature and encompassing multiple research methodologies including basic science, observational, and experimental designs. This scope, together with the intention to organise the review according to the full range of StEP-advocated domains, means that new quantitative secondary analyses or formal quality assessment of all included studies was not undertaken.

In undertaking this review, we chose to limit our search strategy to the previous 5 years. Although a time-limited search scope might be considered a weakness in evidence synthesis exercises, we chose to focus on recent evidence in view of the rapid evolution of surgical techniques and perioperative treatments. This limited search window also allowed a more detailed evaluation of the evidence. To complement our work on neuraxial techniques, we note the recent publication of Admiraal and colleagues,^149^ where outcomes after the use of peripheral regional anaesthesia are considered from the most recent 10-yr period. This work is complementary to that presented here, and we commend it to the reader.

We have not considered cardiac, obstetric, and paediatric surgery separately. Although neuraxial techniques have specific indications and demonstrated benefits in these subgroups, we chose to limit the scope of this work in order to consider the impact of regional anaesthesia on outcomes in the broader population. We acknowledge that a similar exercise is required in special populations. We also acknowledge our search strategy may have failed to identify some relevant scholarly outputs on this topic or that we omitted published works from narrative description because we judged them less impactful than the cited studies. This subjective element of study selection is a recognised weakness of any narrative synthesis. Finally, we have not considered technical complications and direct adverse events arising from neuraxial techniques, although our literature search returned recently published examinations of this important topic.^150^

To conclude, we have summarised extensive research published in the last 5 years into the possible impact of neuraxial techniques on patient outcomes. Evidence of positive impact is best established for domains of patient comfort, pulmonary complications, and mortality—particularly in the setting of major open thoracoabdominal surgery. In contrast, recent evidence does not strongly support a significant impact of neuraxial techniques on cancer, renal, infective, or cardiovascular outcomes after noncardiac surgery in most patient groups. Future clinical trials and subsequent meta-analyses on neuraxial techniques will benefit from more structured inclusion of StEP-advocated patient outcomes.

## Authors’ contributions

Review design and planning: DWH, JGH

Review conduct, writing and revision of manuscript: all authors

## Declarations of interest

DWH and JGH accept fees for advising in civil, criminal, and coronial medicolegal matters. DWH and TT are members of the associate editorial board of the *British Journal of Anaesthesia*. JGH is associate editor-in-chief of the *British Journal of Anaesthesia*.
