# Supplementary material for: Impact of spinal or epidural anaesthesia on perioperative outcomes in adult noncardiac surgery: a narrative review of recent evidence
Source: Br J Anaesth. 2024 May 28;133(2):380–99. doi: 10.1016/j.bja.2024.04.044 (PMC11282476; doi:10.1016/j.bja.2024.04.044)
Supplement: Multimedia component 1 [file mmc1.docx]

1. Search strategy: General and specialty journals included in search

**Acta anaesthesiologica Scandinavica**

Nordisk anaestesiologisk forening; Scandinavian Society of Anaesthesiology and Intensive Care Medicine.

NLM Title Abbreviation: Acta Anaesthesiol Scand

ISSN:0001-5172 (Print) ; 1399-6576 (Electronic) ; 0001-5172 (Linking)

Oxford, UK : Wiley-Blackwell

Currently indexed for MEDLINE

NLM ID: 0370270 [Serial]

**Anaesthesia, critical care & pain medicine**

Société française d'anesthésie et de réanimation.

NLM Title Abbreviation: Anaesth Crit Care Pain Med

ISSN 0750-7658

Masson SAS on behalf of the Société française d'anesthésie et de réanimation (Sfar), [2015]-

NLM ID: 101652401 [Serial]

**Anaesthesia**

Association of Anaesthetists of Great Britain and Ireland.

NLM Title Abbreviation: Anaesthesia

ISSN:0003-2409 (Print) ; 1365-2044 (Electronic) ; 0003-2409 (Linking)

Oxford, UK : Wiley-Blackwell

Currently indexed for MEDLINE

NLM ID: 0370524 [Serial]

**Anesthesia and analgesia**

International Anesthesia Research Society.

NLM Title Abbreviation: Anesth Analg

ISSN:0003-2999 (Print) ; 1526-7598 (Electronic) ; 0003-2999 (Linking)

1998- : Baltimore, Md. : Lippincott Williams & Wilkins

Currently indexed for MEDLINE

NLM ID: 1310650 [Serial]

**Anesthesiology**

American Society of Anesthesiologists; American Society of Anesthetists.

NLM Title Abbreviation: Anesthesiology

ISSN:0003-3022 (Print) ; 1528-1175 (Electronic) ; 0003-3022 (Linking)

Philadelphia Pa : Lippincott Williams & Wilkins

Currently indexed for MEDLINE

NLM ID: 1300217 [Serial]

**Best practice & research. Clinical anaesthesiology**

NLM Title Abbreviation: Best Pract Res Clin Anaesthesiol

Title(s):

ISSN 1521-6896

Amsterdam : Harcourt Publishers, c2001-

ISSN: 1753-3740 (Print) 1878-1608 (Electronic) 1753-3740 (Linking)

Currently indexed for MEDLINE.

NLM ID:101121446 [Serial]

**BMC anesthesiology**

NLM Title Abbreviation: BMC Anesthesiol

[London] : BioMed Central, 2001-

ISSN: 1471-2253 (Electronic) 1471-2253 (Linking) LCCN:2001243457

Currently indexed for MEDLINE.

NLM ID: 100968535 [Serial]

**British journal of anaesthesia**

NLM Title Abbreviation: Br J Anaesth

ISSN:0007-0912 (Print) ; 1471-6771 (Electronic) ; 0007-0912 (Linking)

2018- : [London] : Elsevier

Currently indexed for MEDLINE

NLM ID: 0372541 [Serial]

**Canadian journal of anaesthesia = Journal canadien d'anesthésie**

Canadian Anaesthetists' Society; Canadian Anesthesiologists' Society.

NLM Title Abbreviation: Can J Anaesth

ISSN:0832-610X (Print) ; 1496-8975 (Electronic) ; 0832-610X (Linking)

New York : Springer New York

Currently indexed for MEDLINE

NLM ID: 8701709 [Serial]

**The Clinical journal of pain**

American Academy of Pain Medicine.

NLM Title Abbreviation: Clin J Pain

ISSN:0749-8047 (Print) ; 1536-5409 (Electronic) ; 0749-8047 (Linking)

Hagerstown, MD : Lippincott Williams & Wilkins

Currently indexed for MEDLINE

NLM ID: 8507389 [Serial]

**Current opinion in anaesthesiology**

NLM Title Abbreviation: Curr Opin Anaesthesiol

ISSN:0952-7907 (Print) ; 1473-6500 (Electronic) ; 0952-7907 (Linking)

Philadelphia, PA : Lippincott Williams & Wilkins

Currently indexed for MEDLINE

NLM ID: 8813436 [Serial]

**The Cochrane database of systematic reviews**

NLM Title Abbreviation: Cochrane Database Syst Rev

ISSN:1469-493X (Electronic) ; 1361-6137 (Undetermined) ; 1361-6137 (Linking)

In: Cochrane library (Online).

2004-: Chichester, West Sussex, England : Wiley

Currently indexed for MEDLINE

NLM ID: 100909747 [Serial]

**Pain research & management**

Canadian Pain Society.

NLM Title Abbreviation: Pain Res Manag

ISSN:1203-6765 (Print) ; 1918-1523 (Electronic) ; 1203-6765 (Linking)

<2015- > : New York, NY : Hindawi Publishing Corporation

Currently indexed for MEDLINE

NLM ID: 9612504 [Serial]

**European journal of anaesthesiology**

European Academy of Anaesthesiology.

NLM Title Abbreviation: Eur J Anaesthesiol

ISSN:0265-0215 (Print) ; 1365-2346 (Electronic) ; 0265-0215 (Linking)

Cambridge : Lippincott Williams & Wilkins, 2009-

Currently indexed for MEDLINE

NLM ID: 8411711 [Serial]

**European journal of pain**

International Association for the Study of Pain European Federation of Chapters.

NLM Title Abbreviation: Eur J Pain

ISSN:1090-3801 (Print) ; 1532-2149 (Electronic) ; 1090-3801 (Linking)

2012- : Chichester, UK : Wiley

Currently indexed for MEDLINE

NLM ID: 9801774 [Serial]

**International journal of obstetric anesthesia**

Obstetric Anaesthetists' Association.

NLM Title Abbreviation: Int J Obstet Anesth

ISSN:0959-289X (Print) ; 1532-3374 (Electronic) ; 0959-289X (Linking)

<2004->: Amsterdam, The Netherlands : Elsevier

Currently indexed for MEDLINE

NLM ID: 9200430 [Serial]

**JAMA**

American Medical Association.

NLM Title Abbreviation: JAMA

ISSN:0098-7484 (Print) ; 1538-3598 (Electronic) ; 0098-7484 (Linking)

Chicago: American Medical Association, 1960-

Currently indexed for MEDLINE

NLM ID: 7501160 [Serial]

**Journal of anesthesia**

Nihon Masui Gakkai.

NLM Title Abbreviation: J Anesth

ISSN:0913-8668 (Print) ; 1438-8359 (Electronic) ; 0913-8668 (Linking)

Tokyo : Springer International for the Japan Society of Anesthesiology

Currently indexed for MEDLINE

NLM ID: 8905667 [Serial]

**Journal of cardiothoracic and vascular anesthesia**

NLM Title Abbreviation: J Cardiothorac Vasc Anesth

ISSN:1053-0770 (Print) ; 1532-8422 (Electronic) ; 1053-0770 (Linking)

Philadelphia, PA : W.B. Saunders, c1991-

Currently indexed for MEDLINE

NLM ID: 9110208 [Serial]

**Journal of clinical anesthesia**

NLM Title Abbreviation: J Clin Anesth

ISSN:0952-8180 (Print) ; 1873-4529 (Electronic) ; 0952-8180 (Linking)

<2008->: New York : Elsevier

Currently indexed for MEDLINE

NLM ID: 8812166 [Serial]

**Journal of clinical monitoring and computing**

European Society for Computing and Technology in Anaesthesia and Intensive Care; Society for Technology in Anesthesia; American Society of Neurophysiological Monitoring.

NLM Title Abbreviation: J Clin Monit Comput

ISSN:1387-1307 (Print) ; 1573-2614 (Electronic) ; 1387-1307 (Linking)

Amsterdam : Springer

Currently indexed for MEDLINE

NLM ID: 9806357 [Serial]

**The journal of headache and pain**

Società italiana per lo studio delle cefalle; European Headache Federation. NLM

Title Abbreviation: J Headache Pain. ISSN:1129-2369 (Print) ; 1129-2377

(Electronic) ; 1129-2369 (Linking). [2018]- : [London, United Kingdom] : BioMed

Central. Currently indexed for MEDLINE.

NLM ID: 100940562 [Serial]

**Journal of neurosurgical anesthesiology**

NLM Title Abbreviation: J Neurosurg Anesthesiol

ISSN:0898-4921 (Print) ; 1537-1921 (Electronic) ; 0898-4921 (Linking)

Hagerstown, MD : Lippincott Williams & Wilkins

Currently indexed for MEDLINE

NLM ID: 8910749 [Serial]

**The journal of pain**

American Pain Society.

NLM Title Abbreviation: J Pain

ISSN:1526-5900 (Print) ; 1528-8447 (Electronic) ; 1526-5900 (Linking)

Philadelphia, PA : Churchill Livingstone, c2000-

Currently indexed for MEDLINE

NLM ID: 100898657 [Serial]

**Journal of palliative medicine**

American Academy of Hospice and Palliative Medicine; Center to Advance Palliative Care; Hospice and Palliative Nurses Association; ANZSPM (Organization); Nihon Kanwa Iryō Gakkai.

NLM Title Abbreviation: J Palliat Med

ISSN:1096-6218 (Print) ; 1557-7740 (Electronic) ; 1557-7740 (Linking)

Larchmont, NY : Mary Ann Liebert, Inc., c1998-

Currently indexed for MEDLINE

NLM ID: 9808462 [Serial]

**Korean journal of anesthesiology**

Taehan Mach'wikwa Hakhoe; Taehan Mach'wi T'ongchŭg Ŭihakhoe.

NLM Title Abbreviation: Korean J Anesthesiol

ISSN:2005-6419 (Print) ; 2005-7563 (Electronic) ; 2005-6419 (Linking)

Seoul : Korean Society of Anesthesiologists

Currently indexed for MEDLINE

NLM ID: 101502451 [Serial]

**Lancet (London, England)**

NLM Title Abbreviation: Lancet

ISSN:0140-6736 (Print) ; 1474-547X (Electronic) ; 0140-6736 (Linking)

2004- : London : Elsevier

Currently indexed for MEDLINE

NLM ID: 2985213R [Serial]

**Minerva anestesiologica**

Società Italiana di Anestesia Analgesia Rianimazione e Terapia Intensiva; Società italiana di anestesiologia; Società Italiana di Anestesia e Rianimazione.

NLM Title Abbreviation: Minerva Anestesiol

ISSN:0375-9393 (Print) ; 1827-1596 (Electronic) ; 0375-9393 (Linking)

Torino, [Edizioni Minerva Medica]

Currently indexed for MEDLINE

NLM ID: 0375272 [Serial]

**Molecular pain**

NLM Title Abbreviation: Mol Pain

ISSN:1744-8069 (Electronic) ; 1744-8069 (Linking)

2016- : Thousand Oaks, CA : Sage Publications Inc.

Currently indexed for MEDLINE

NLM ID: 101242662 [Serial]

**The New England journal of medicine**

Aberman, Arnold; Massachusetts Medical Society.

NLM Title Abbreviation: N Engl J Med

ISSN:0028-4793 (Print) ; 1533-4406 (Electronic) ; 0028-4793 (Linking)

Boston, Massachusetts Medical Society.

Currently indexed for MEDLINE

NLM ID: 0255562 [Serial]

**Nature medicine**

NLM Title Abbreviation: Nat Med

ISSN:1078-8956 (Print) ; 1546-170X (Electronic) ; 1078-8956 (Linking)

New York Ny : Nature Publishing Company

Currently indexed for MEDLINE

NLM ID: 9502015 [Serial]

**Neuromodulation: journal of the International Neuromodulation Society**

International Neuromodulation Society.

NLM Title Abbreviation: Neuromodulation

ISSN:1094-7159 (Print) ; 1525-1403 (Electronic) ; 1094-7159 (Linking)

2022- : [New York] : Elsevier

Currently indexed for MEDLINE

NLM ID: 9804159 [Serial]

**Pain**

International Association for the Study of Pain.

NLM Title Abbreviation: Pain

ISSN:0304-3959 (Print) ; 1872-6623 (Electronic) ; 0304-3959 (Linking)

2015- : Hagerstown, MD : Lippincott Williams & Wilkins

Currently indexed for MEDLINE

NLM ID: 7508686 [Serial]

**Pain management**

NLM Title Abbreviation: Pain Manag

ISSN:1758-1869 (Print) ; 1758-1877 (Electronic) ; 1758-1869 (Linking)

London : Future Medicine

Currently indexed for MEDLINE

NLM ID: 101555934 [Serial]

**Pain medicine**

American Academy of Pain Medicine.

NLM Title Abbreviation: Pain Med

ISSN:1526-2375 (Print) ; 1526-4637 (Electronic) ; 1526-2375 (Linking)

2016- : Oxford, England : published by Oxford University Press on behalf of the American Academy of Pain Medicine

Currently indexed for MEDLINE

NLM ID: 100894201 [Serial]

**Pain physician**

Association of Pain Management Anesthesiologists; American Society of Interventional Pain Physicians.

NLM Title Abbreviation: Pain Physician

ISSN:1533-3159 (Print) ; 2150-1149 (Electronic) ; 1533-3159 (Linking)

Paducah, Ky. : American Society of Interventional Pain Physicians

Currently indexed for MEDLINE

NLM ID: 100954394 [Serial]

**Pain practice**

World Institute of Pain.

NLM Title Abbreviation: Pain Pract

ISSN:1530-7085 (Print) ; 1533-2500 (Electronic) ; 1530-7085 (Linking)

Malden, Mass. : Blackwell Science, Inc., c2001-

Currently indexed for MEDLINE

NLM ID: 101130835 [Serial]

**Pain research & management**

Canadian Pain Society.

NLM Title Abbreviation: Pain Res Manag

ISSN:1203-6765 (Print) ; 1918-1523 (Electronic) ; 1203-6765 (Linking)

<2015- > : New York, NY : Hindawi Publishing Corporation

Currently indexed for MEDLINE

NLM ID: 9612504 [Serial]

**Palliative medicine**

NLM Title Abbreviation: Palliat Med

ISSN:0269-2163 (Print) ; 1477-030X (Electronic) ; 0269-2163 (Linking)

London : SAGE Publications

Currently indexed for MEDLINE

NLM ID: 8704926 [Serial]

**Regional anesthesia and pain medicine**

American Society of Regional Anesthesia.

NLM Title Abbreviation: Reg Anesth Pain Med

ISSN:1098-7339 (Print) ; 1532-8651 (Electronic) ; 1098-7339 (Linking)

2018- : [London, United Kingdom] : BMJ

Currently indexed for MEDLINE

NLM ID: 9804508 [Serial]

Search strategy: Terms

| Ovid Search Details applied to journals |
| --- |
| 1. “spinal”.mp  2. “intrathecal”.mp  3. “subarachnoid”.mp  4. “epidural”.mp  5. “extradural”.mp  6. “caudal”.mp  7. “neuraxial”.mp  8. 1 or 2 or 3 or 4 or 5 or 6 or 7  9. “analges*”.mp  10. “anaesth*”.mp  11. “anesth*”.mp  12. 9 or 10 or 11  13. 8 and 12  14. limit 13 to (yr=2018/01/01-2023/09/01)  15. limit 14 to (English language) |
